# Soil nematode functional diversity, successional patterns, and indicator taxa associated with vertebrate decomposition hotspots

**DOI:** 10.1371/journal.pone.0241777

**Published:** 2020-11-04

**Authors:** Lois S. Taylor, Gary Phillips, Ernest C. Bernard, Jennifer M. DeBruyn

**Affiliations:** 1 Department of Biosystems Engineering and Soil Science, University of Tennessee, Knoxville, Tennessee, United States of America; 2 Department of Entomology and Plant Pathology, University of Tennessee, Knoxville, Tennessee, United States of America; University of Illinois at Urbana-Champaign, UNITED STATES

## Abstract

Decomposition of vertebrate remains is a dynamic process that creates localized soil enrichment zones. A growing body of literature has documented effects of vertebrate decomposition on soil pH, electrical conductivity, oxygen levels, nitrogen and carbon speciation, microbial biomass, and microbial successional patterns. However, relatively few studies have examined the microfaunal members of the soil food web that function as secondary consumers, specifically nematodes. Nematodes are often used as indicators of enrichment in other systems, and initial observations from vertebrate decomposition zones have indicated there is an effect on nematode communities. Our goal was to catalog decomposition-induced nematode succession and changes to alpha, beta, and functional diversity, and identify potential indicator taxa associated with decomposition progression. Six adult beaver (*Castor canadensis*) carcasses were allowed to decompose in a forest ecosystem for one year. During this period soil temperature, moisture, and electrical conductivity were monitored. Soils samples were taken at two depths in order to assess nematode community dynamics: 30-cm cores and 1-cm interface samples. Nematode abundance, alpha, beta, and functional diversity all responded to soil enrichment at the onset of active decay, and impacts persisted through skeletonization. After one year, nematode abundances and alpha diversity had recovered to original levels, however both community membership and functional diversity remained significantly altered. We identified seven indicator taxa that marked major transitions in decomposition progression. Enrichment of Rhabditidae (B1) and Diplogasteridae (B1) coupled with depletion in *Filenchus* (F2) characterized active and advanced decay prior to skeletonization in both cores and interface soils. Enrichment of *Acrobeloides* (B2), *Aphelenchoides* (F2), Tylencholaimidae (F4) and *Seinura* (P2) occurred during a narrow period in mid-skeletonization (day 153). Our study has revealed soil nematode successional patterns during vertebrate decomposition and has identified organisms that may function as indicator taxa for certain periods during decomposition.

## Introduction

Decomposition of vertebrate remains is a dynamic process that creates localized soil enrichment zones. These biogeochemical hotspots exhibit increased rates of nutrient cycling which are short-lived in comparison with surrounding areas [1,2]. There is a growing body of literature documenting the effects of vertebrate decomposition on soil in these hotspots, which include a wide range of abiotic, biotic, and biotically-induced parameters. Soil chemistry studies have revealed temporally-associated changes in pH [2–11], electrical conductivity [5,8,10,11], soil oxygen levels [2], enzyme profiles [2], fatty acid residues [12], steroid fingerprints [13], and fluxes in carbon and nitrogen speciation pools [2,3,5–8,10,11]. High-throughput sequencing has been used to examine bacterial (and to a lesser extent, fungal) community composition and successional patterns [6,7,14–19]. However, in comparison with soil chemistry and microbial studies, targeted examination of multicellular soil faunas, particularly microfaunal members of the soil food web that function as secondary consumers of these resources, have received the least attention [but see 20–24].

Microscopic soil animals, or microfauna, is a designation that includes tardigrades, mites, rotifers, and nematodes, among others. Of these microfauna, nematodes are estimated to comprise some 80% of all multicellular organisms found in the soil environment [25]. Nematodes are well-studied across a wide range of environments, ecosystems, and biomes, at scales ranging from local to global [26–28]. They can be divided into morphologically distinct trophic groups (bacterial feeders, fungal feeders, plant feeders, omnivores, and predators), and thus exhibit functionally-partitioned responses to environmental changes [29]. These trophic groups can be further subdivided according to colonizer-persister (cp-) classifications, based upon an *r-* and *K*-selection continuum [30]. Thus, nematode community diversity and successional patterns have been shown to be sensitive indicators of soil enrichment and disturbance in agricultural systems [31–43] and composting processes [44,45]. Many field studies of nematodes focus on the upper 30 cm of soil, where the majority of free-living nematodes reside [24,31,35–41,43,46–49]. Vertical and horizontal distributions of soil nematodes are most commonly driven by food source location [47,48]; nematode density, particularly those of bacterivores, correlates with areas enriched in soil organic carbon (SOC), and therefore higher densities of bacterial biomass [28].

Nematode community dynamics resulting from soil enrichment during vertebrate decomposition have been only initially explored [24]. Szelecz et al. (2016) observed successional patterns of nematode community diversity during pig carcass decomposition which were similar to patterns observed in composting and amended agricultural soils. While the study by Szelecz et al. (2016) [24] showed changes in community membership at the family level, they did not report changes in taxa absolute abundance. Given the substantial differences in ecological responses between cp classes to ecosystem disturbances, it is likely that a finer, or perhaps mixed, taxonomic resolution is needed to document patterns and identify potential marker taxa linked with decomposition progress. Data derived from human and animal vertebrate decomposition studies have demonstrated that seasonality, climate, and choice of decomposing organism all affect the rates and patterns of decomposition progression [4,50,51]. Taken together, these studies highlight both the potential for using nematode community changes as temporal markers to track decomposition progress, as well as a critical need to better characterize nematode successional and functional diversity dynamics during decomposition across a wide range of decomposing organisms as well as environmental and edaphic conditions.

Our study was designed to build upon existing findings and fill a gap in our understanding of microfaunal dynamics associated with vertebrate decomposition. Our goal was to catalog decomposition-induced nematode succession and changes to functional diversity with fine resolution, both temporally and taxonomically, in soils beneath decomposing vertebrate carcasses. We expected to observe successional and functional diversity changes in nematode community composition through time commensurate with the progression of decomposition of vertebrate carcasses. We expected to detect potential indicator taxa that were both positively and negatively correlated to the conditions present in these hotspot environments over extended time periods. Lastly, we hypothesized that the interface between the decomposing vertebrate material and the top-most organic layer of soil would harbor a more distinctive nematode community succession than deeper in the soil profile.

## Materials and methods

### Experimental site

This study took place over the course of 11 months, spanning March 2017 through February 2018, and was conducted at the Oak Ridge Arboretum, part of the University of Tennessee Forest Resources AgResearch and Education facilities, located in Oak Ridge, TN, USA (35° 59’ 36.28” N, 84° 13’ 12.34” W). The climate is categorized as *Cfa* (Humid subtropical) according to the Köppen classification system. Mean annual precipitation is 143 cm; mean annual air temperature is 13.6°C. Temperatures during this study ranged between 3°C and 35°C, with a mean of 19.7°C [52]. Soils belong to the Fullerton-Pailo complex, comprised of approximately 60% Fullerton and 30% Pailo soils, with a parent material of cherty limestone. The ecological site listing is: thermic cherty dolomite upland oak-hickory forest [53]. Beavers were selected for this experiment as a model of a local, mid-sized, wild vertebrate. Nuisance beaver carcasses (*Castor canadensis*) were obtained from East Tennessee USDA. All animals used in this study were salvaged carcasses, therefore IACUC approval was not required. Carcasses were stored at -20°C and placed frozen at the experimental site at the start of the experiment. Six mature adult beavers were used for this study, approximately 20 to 23 kg each. Beavers were placed in wire cages to allow contact with soil and access by insect scavengers, while eliminating animal and avian scavenging. Cages were placed 3 m apart. Sensors were placed in the soil underneath each carcass to measure soil temperature, moisture, and electrical conductivity (Decagon Devices, GS3). Temperature probes were inserted rectally into each carcass 1 day after placement (Decagon Devices, RT-1). Soil and internal sensors were removed late in the decay process after temperatures returned to ambient (day 100 of the study).

### Soil sampling and nematode extraction

Soil samples were taken at twelve time points throughout decomposition. The first six samples corresponded to visual assessments of decomposition stages after Payne (1965) and Cobaugh et al. (2015) [6,54]: Placement, day 0; fresh, day 2; bloat, day 6; active decay, day 15; advanced decay 1, day 21; advanced decay 2, day 40. After skeletonization, sampling continued on a monthly basis for four months (days 78, 110, 153, 188), and at roughly two-month intervals thereafter (days 250, 317). At each sampling time point three 30-cm cores were removed from underneath each of the six carcasses with a 1.9 cm (¾-inch) diameter soil probe, and composited to generate one sample per carcass, for a total of six samples per time point. Soils were also sampled from paired control sites located approximately 4 m from each carcass that were unimpacted by decomposition, for a total of six control soil samples at each sampling time point. Early results of nematode response to cadaver-enrichment suggested stratification in the soil column. Therefore, starting at day 15, we additionally collected the top 1 cm of soil from directly underneath carcasses. This 0–1 cm sample was referred to as an interface sample and was included to explore hotspot effects in immediate proximity to decomposing material. Care was taken in order not to sample the same areas twice. Samples were immediately transferred to the laboratory and stored at 4°C until nematode extraction.

Nematodes were extracted from soil by means of a sugar-flotation centrifugation technique as described by Jenkins (1964) [55]. All extractions were performed by the same person. Total abundances were expressed as nematodes per 100 cm^3^ soil. Nematodes were counted and identified from live samples by differential interference contrast (DIC) microscopy. Taxa were identified primarily to genus level, and to family or order level where appropriate.

### Alpha, beta, and functional diversity

Nematode communities were characterized as described in Keenan et al. (2018) [56]. Briefly, nematodes were assigned trophic groups, colonizer-persister (cp) classifications, and functional guilds using methods described previously [29,30,33]. *Filenchus* was classified as an F2 fungivore according to Okada et al. (2005) [57]. The family Tylenchidae is very large and currently not well studied, and frequently Tylenchidae ecology is based upon observations of resource proximity rather than cultured studies [58,59]. *Filenchus*, *Lelenchus*, and *Malenchus*, have recently been shown to be closely related and thus for the purposes of this study both *Lelenchus* and *Malenchus* have been classified as F2 fungivores [58]. *Ecphyadophora* has no clearly defined ecological function in the literature, however it also has been shown to be closely related to *Malenchus*; according to similar classification and behavioral characteristics *Ecphyadophora* has thus been classified as an F4 fungivore [58,59]. Richness was defined as the number of unique taxa at family and genus levels. Shannon diversity was calculated as described in Keenan et al. (2018) [56]. Functional diversity indices were calculated according to Ferris et al. (2001) and Keenan et al. (2018) [56,60]. All visualizations were created in R (version 3.6.1) using the ggplot2 (version 3.2.1), RColorBrewer (version 1.1–2), and pheatmap (version 1.0.12) packages [61–64].

### Statistical analysis

The six carcass sites in this study were treated as experimental replicates. Six paired control sites, one associated with each carcass, were treated as control replicates. Two-way ANOVA (p < 0.05) was conducted to test for significant differences between the effects of decomposition products and sampling time point. This was followed by paired T-tests at each sampling time point to determine if decomposition-impacted soils significantly differed from control soils. Redundancy analysis (RDA) on Euclidean distances was performed in order to visualize community differences by decomposition stage, as well as between core and interface soils as a whole. Ellipses represent standard deviations from decay stage centroids. Analyses were conducted in R (version 3.6.1) using the tidyverse (version 1.2.1), car (version 3.0–7), and vegan packages [63,65–67].

### Identification of indicator taxa

Due to ranges in r- and *K*-selection characteristics present in each trophic group, variations in taxonomic composition of cp-classes, and acknowledging the limitations of cp- class designations (whereby membership of the same cp-class consists of multiple organisms whose life-histories are not always well-documented), care must be taken in assigning indicator taxa in order to avoid inferring cross-taxonomic or life-history relationships that might not exist. To assess candidacy of an individual taxon as an indicator, both positive (enrichment) and negative (suppression) response patterns were determined using the following criteria. Nematode taxa were identified as exhibiting enrichment if they satisfied two criteria: 1) a minimum mean abundance of five at any sampling time point (study day); and 2) increase above a threshold, which was calculated by multiplying the mean abundance in the control soils by a scaling factor (10 for B1 organisms, two for all others). The satisfaction of both criteria screened for well-represented taxa that exhibit a substantial increase and eliminated rare taxa whose overall sample representation would be low regardless of enrichment. Nematode taxa exhibiting suppression were also required to satisfy two simultaneous criteria: 1) a minimum mean abundance of five in controls, and 2) sample decrease to below a threshold, which was calculated by dividing the control mean by the same scaling factor as used for enrichment criteria (10 for B1 organisms, two for all others). The satisfaction of both criteria screened for initially well-represented taxa that exhibited a substantial decrease, and eliminated rare taxa that exhibited uneven sample representation, and therefore would not be considered robust indicators. Scaling factors of 10 for B1 enrichment opportunists and two for all other taxa were selected based upon ecological patterns. Thresholds of five were chosen in order to exclude rare taxa whose appearance in samples could not be consistently observed or demonstrate robust response patterns. Core (0–30 cm) and interface (0.1 cm) soils were assessed separately.

In order to visualize overall response patterns as well as indicator taxon patterns, heatmaps were constructed for both core (0–30 cm) and interface (0–1 cm) soils and subdivided by trophic group (bacterivores, fungivores, omnivores, and predators). Each trophic group heatmap was then organized according to functional guilds (cp-class) (B1, B2, etc.) in descending order from greatest r-selection to greatest K-selection. Within each cp-class taxa were alphabetized. All taxa within the above trophic categories are included regardless of numerical contribution.

## Results

### Decomposition stages

**Fig 1:**
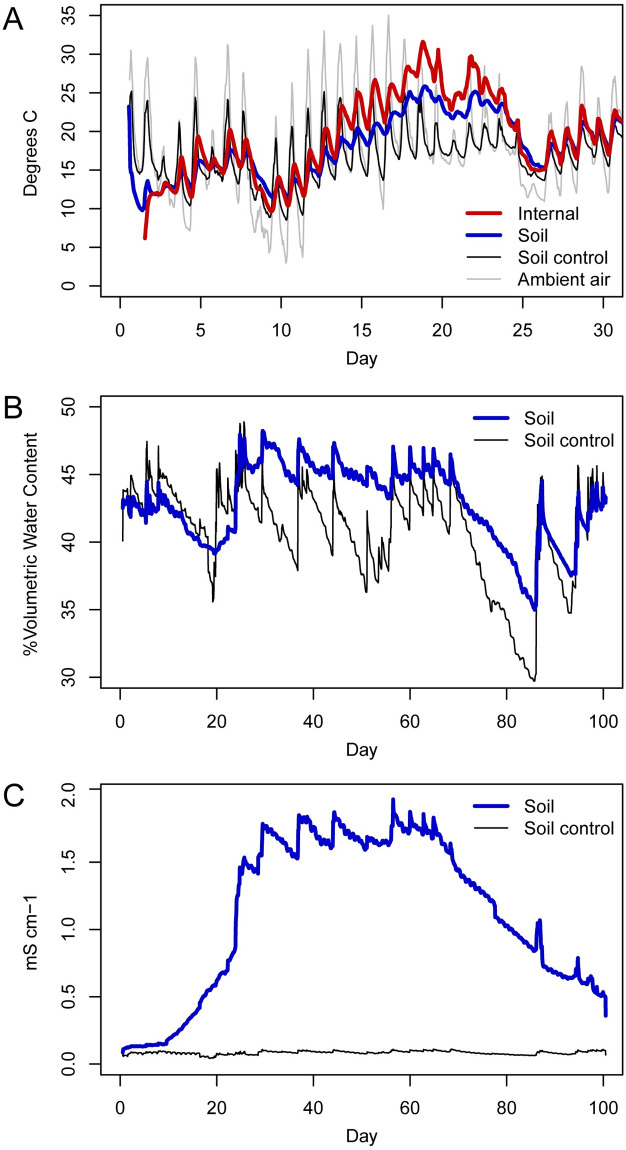
Soil and ambient environmental sensor data. (A) Mean internal, ambient, and soil temperatures, (B), mean soil moisture, and (C) mean soil electrical conductivity. Internal carcass temperature increased beginning on day 13 and remained elevated until day 25. Soil moisture and electrical conductivity rapidly increased following day 21 and remained elevated until day 95. Decomposition-impacted soils are shown in blue (A-C), soil controls are shown in black (A-C), internal temperatures are shown in red (A), and ambient air temperatures are shown in gray (A).

Six frozen beaver carcasses were placed at the experimental site on day 0. By day 1 carcasses had thawed sufficiently that internal temperature probes could be inserted rectally; external temperatures of carcasses had equilibrated by this time; however, core body temperatures remained below ambient air temperature. By day 2 internal temperatures had equilibrated with ambient air temperature, and carcasses were considered to be at the fresh stage. Fly egg masses were visible in the eyes. Early bloating occurred by day 6. Bloating continued for nine days, in part due to a period of colder weather (Fig 1A). During the bloat period the main body portion of the carcasses was swollen and skin surfaces could not be depressed when touched. Maggot masses were present in the facial and rectal areas, with no visible seepage of fluids from those areas. Active decay, day 15, was marked by an increase in maggot activity in the torso area, the movement of decomposition fluid into the soil and the initial formation of visible cadaver decomposition islands (CDIs). Advanced decay began at day 21, after the majority of tissue mass was gone and maggot activity had decreased. Sampling occurred twice during advanced decay: Upon decrease of maggot activity at day 21 (advanced decay 1), and again as greater amounts of remaining tissue were lost but prior to dry and skeletonized remains at day 40 (advanced decay 21). Early skeletonization consisted of bone with some greasy or dry tissue; this stage was sampled twice at monthly intervals: Day 78 (early skeletonization 1) and day 110 (early skeletonization 2). After this period only fully skeletonized remains were left; these were sampled every two months at four time points: Days 153, 188, 250, and 317 (late skeletonization 1–4, respectively) (Table 1).

**Table 1:** Study day, sampling date, and morphological decomposition stage. Decomposition stages that include more than one sampling time point are numbered sequentially.

Study day	Sampling date	Decomposition stage
0	29-Mar-17	Placement
2	31-Mar-17	Fresh
6	4-Apr-17	Bloat
15	13-Apr-17	Active decay
21	19-Apr-17	Advanced decay 1
40	8-May-17	Advanced decay 2
78	15-Jun-17	Early skeletonization 1
110	17-Jul-17	Early skeletonization 2
153	29-Aug-17	Late skeletonization 1
188	3-Oct-17	Late skeletonization 2
250	4-Dec-17	Late skeletonization 3
317	9-Feb-18	Late skeletonization 4

### Internal carcass temperature, soil temperature, moisture, and electrical conductivity

The temperatures of the carcass and decomposition-impacted soils began to increase above ambient air and control soil temperatures during the transition from bloat to active decay (day 13) and remained elevated until day 25, corresponding to the periods of active and early advanced decay and peak maggot activity (Fig 1A). Maximum internal temperatures ranged from 28.0 to 42.4°C with a mean of 31.6°C. Maximum temperatures of impacted soils underneath carcasses ranged from 25.3 to 34.5°C with a mean maximum of 25.8°C. Soil temperatures at control sites did not appreciably deviate from ambient air temperature; however, as expected for soils, the range of diurnal variation in soil was attenuated compared to ambient air temperatures. The maximum temperature of control soils ranged from 26.1 to 27.5°C with a mean maximum of 25.8°C; in comparison, ambient air reached a temperature maximum of 35°C.

Soil moisture rapidly increased following day 21 (primarily in in carcasses 1 and 6), and remained elevated until day 95, corresponding to advanced decay and early skeletonization. During this period, the soil was covered with tissue and fur, and small pockets of adipocere developed. As tissue and fur continued to degrade during the latter portion of advanced decay, soil surfaces were gradually uncovered and soil moisture content decreased. During this period of enhanced soil moisture the mean moisture maximum in impacted soils reached 48.2 percent volumetric water content (%VWC) and individual maxima ranged from 45.2 to 60.0%VWC, whereas control soil maxima ranged from 46.3 to 51.0 with a mean maximum of 48.9%VWC (Fig 1B).

Electrical conductivity began to increase between days 10–20, during bloat (carcass 1) and active decay (all others) as purge decomposition fluids moved into the soil. Conductivity in impacted soils remained elevated throughout early skeletonization, reaching a mean maximum of 2.0 mS cm^-1^ during advanced decay, with individual maxima ranging from 1.3–3.9 mS cm^-1^. Control soil maxima ranged from 0.1–0.2 with a mean maximum of 0.1 mS cm^-1^ (Fig 1C).

### Nematode total abundances and alpha diversity in core (0–30 cm) samples

**Fig 2:**
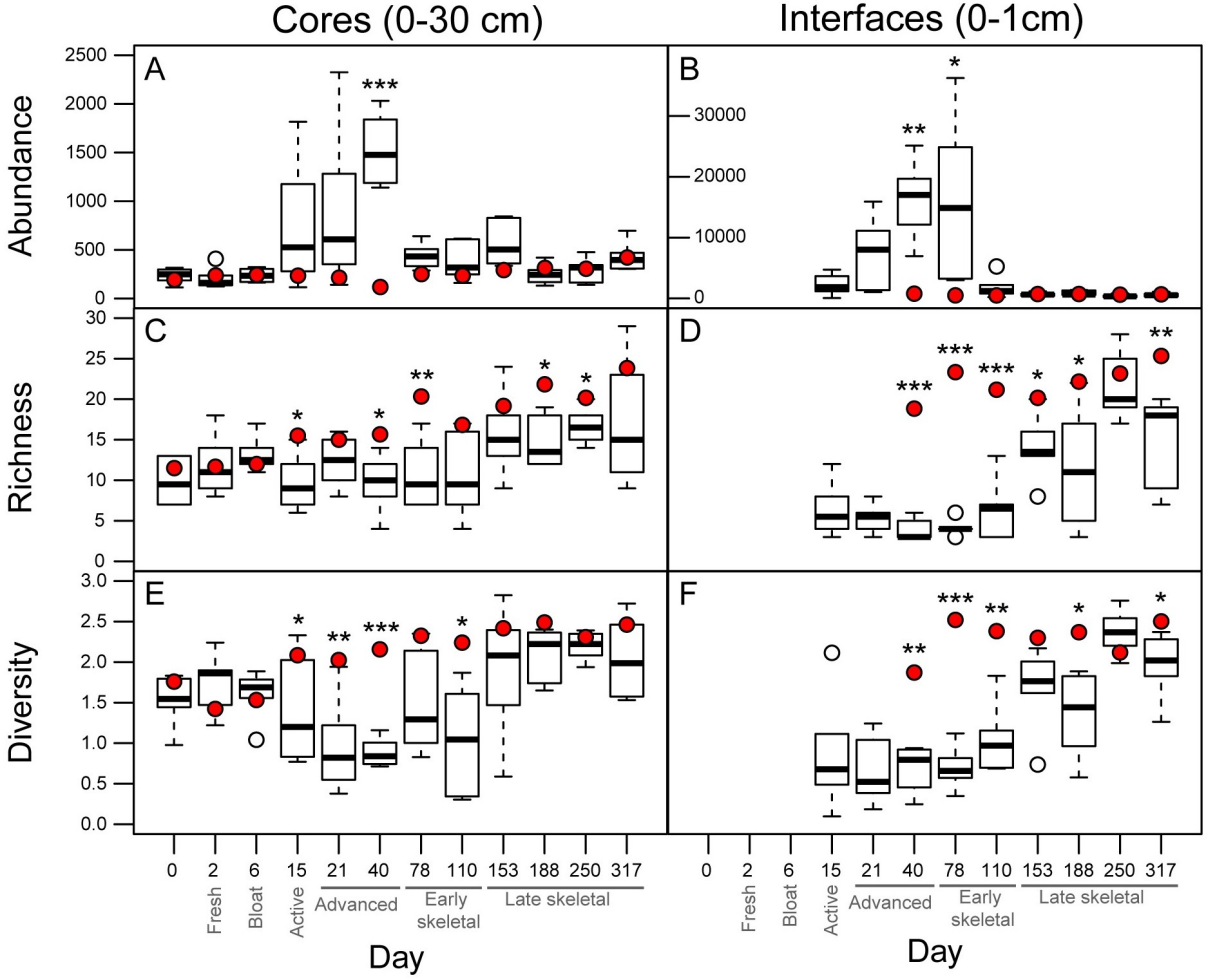
Nematode abundance, richness, and diversity. (A-B) Nematode abundances, (C-D) richness, and (E-F) Shannon diversity (E-F) found in core (0–30 cm) and interface (0–1 cm) soils. Control means are represented by red dots. Interface data collection began on day 15, and interface control data collection began on day 40. Boxes display 25th and 75th quartiles and medians; whiskers display ± 1.5 quartiles.

In decomposition-impacted soils, all nematode indices (abundances, richness, diversity) differed significantly from control soils (p < 0.001) over the course of the study (Fig 2). At the onset of active decay (day 15), nematode abundances began to increase while both richness and diversity decreased. Mean abundances tripled in affected soils between bloat and active decay (days 6–15), while mean richness and diversity decreased by 26.5% and 12.5% respectively. All three indices exhibited mean maxima (abundance) or minima (richness and diversity) on day 40 (advanced decay 2); mean abundances were enriched by 13 times those of control means, whereas richness and diversity had decreased by 38.2% and 60.1%, respectively, compared to their respective controls. Changes in nematode abundance and diversity continued through day 153 (late skeletonization 1) before returning to initial levels, whereas richness remained depressed to the end of the study (Fig 2A, 2C and 2E, S1 and S2 Tables).

### Nematode total abundances and alpha diversity in interface (0–1 cm) samples

Interface soils were collected starting in active decay (day 15) in order to ascertain the degree of nematode activity in the upper-most layer of soil directly in contact with decomposing remains. As with 0–30 cm core samples, all interface (0–1 cm) indices (abundances, richness, and diversity) were observed to significantly differ from controls (p < 0.001) over the course of the entire study.

On days 15 and 21, corresponding to active decay and advanced decay 1, mean nematode abundances in impacted interfaces (0–1 cm) were 3 and 8.6 times higher than found in deeper soil (0–30 cm) samples. Similar to the pattern seen in cores, mean interface maxima (abundances) and minima (richness) occurred on day 40 (advanced decay 2). Mean abundances in interface soils from day 40 were enriched 20.8 times that found in interface controls, and 10.7 times core abundance maxima from the same sampling date. Richness in interfaces on day 40 decreased 79.8% with respect to controls, and 60.1% with respect to the richness minimum found in cores on the same date. Diversity reached a minimum on day 21 (advanced decay1). Overall, interface soils were observed to have increased abundances between days 40–110 (advanced decay through early skeletonization) before returning to initial levels, whereas the impact upon richness and diversity remained throughout the end of the study (Fig 2B, 2D and 2F, S1 and S2 Tables).

### Nematode beta diversity—Community structure in core (0–30 cm) samples

**Fig 3:**
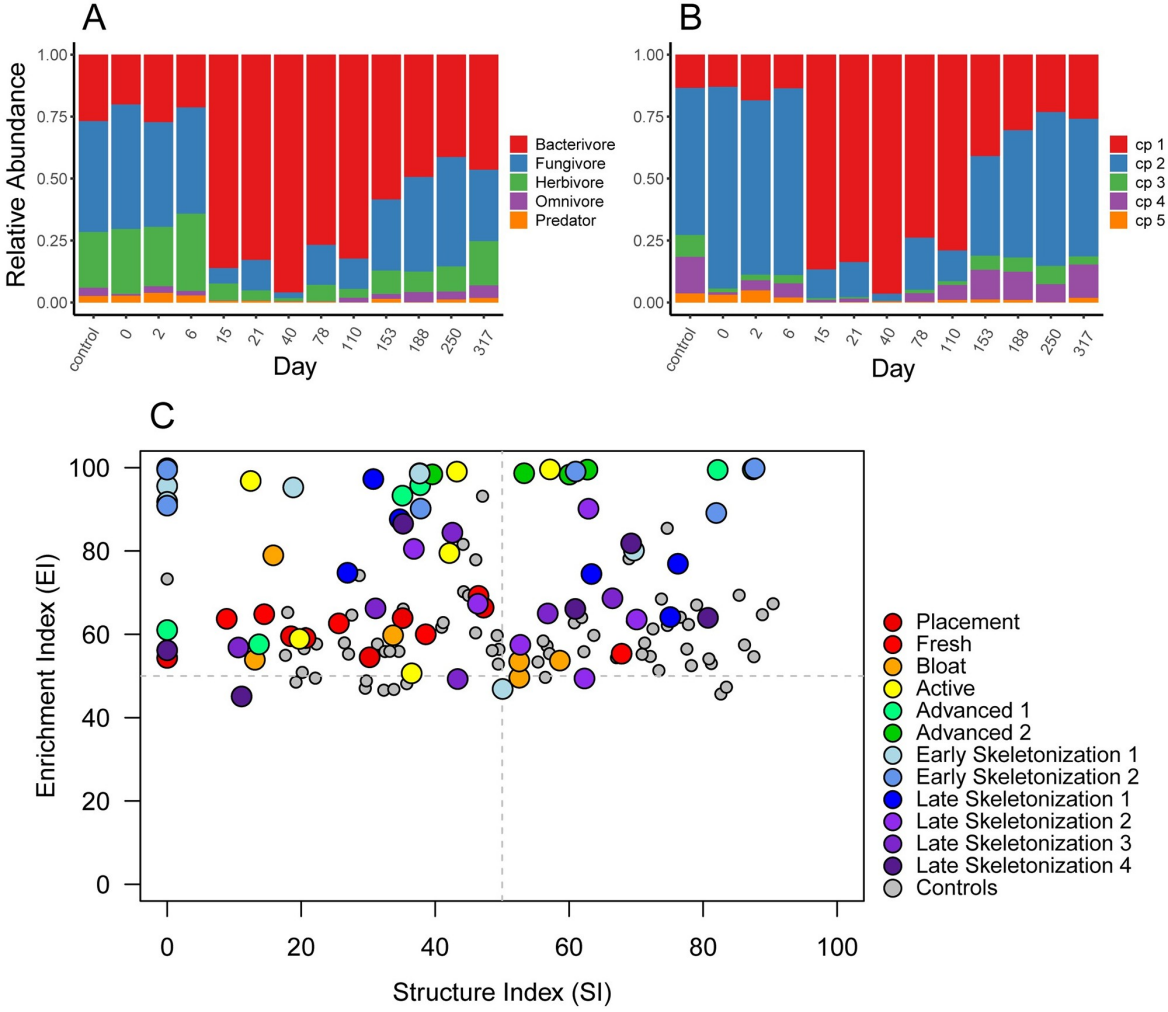
Temporal changes in nematode community composition and functional diversity in core (0–30 cm) soils. Relative abundances of (A) trophic groups, (B) cp-classes, and (C) functional diversity analysis for core (0–30 cm) soils.

**Fig 4:**
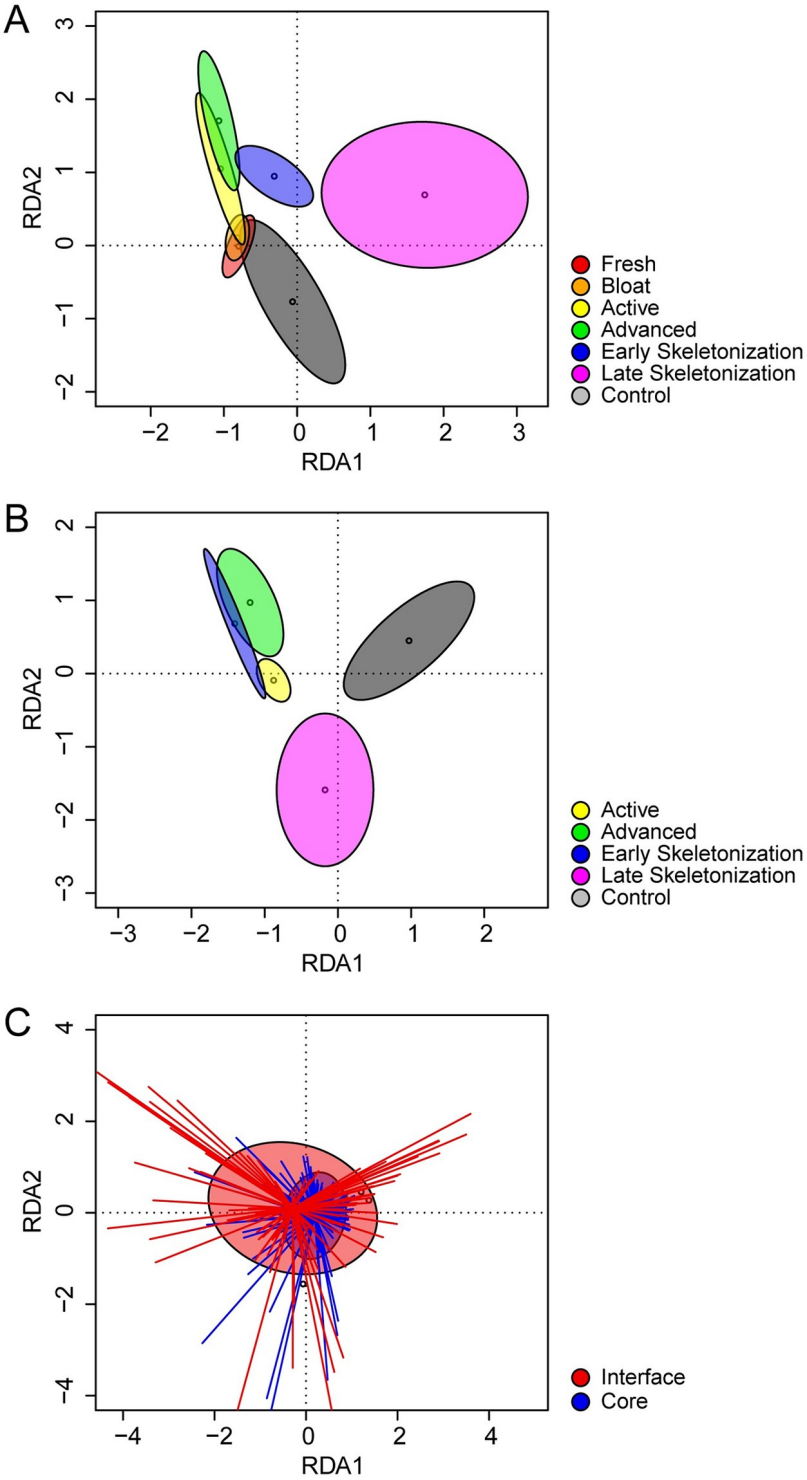
Nematode beta diversity. RDA biplots of nematode community trajectory during decomposition progression in (A) core (0–30 cm) and (B) interface (0–1 cm) soils. Ellipses represent standard deviations from decay stage centroids. Samples are grouped by morphological decomposition stage. Comparative changes at both soil depths (C) are shown for the entire study. Ellipses represent standard deviations from depth centroids, and spiders show distances from centroids.

Fungivores were the primary trophic group found initially in soils at this site. At the onset of active decay (day 15), bacterivores began to proliferate in decomposition-impacted soils, peaking in abundance during advanced decay (day 40), concurrent with the changes observed in abundances, richness, and diversity. After day 40, relative abundances of bacterivores decreased, although not to initial levels: soils were still shifted toward a bacterivore-dominated community after one year (Fig 3A). Temporal changes in colonizer-persister (cp) class composition mirrored patterns found in trophic group succession, shifting from a mixture of largely cp-2 class taxa, to communities dominated by select cp-1 class taxa (an exclusively bacterial feeding designation) at day 15. After day 40 a gradual decrease in cp-1 and concomitant increase in cp-2 through cp-5 class taxa was observed during skeletonization; however, by the end of the study soils were still moderately enriched with cp-1 bacterivores, indicating that communities continued to be impacted by decomposition products (Fig 3B). These shifts in functional diversity were reflected in the faunal profile (Fig 3C). Samples from controls, initial, and bloat decomposition stages populate the centerline of the Enrichment Index (EI), indicative of a mixed bacterial and fungal community composed primarily of cp-2 class taxa with low representation of cp-1 bacterial feeders. During advanced decay, and continuing through early skeletonization, communities populate the upper regions of the EI, indicative of a strong shift towards bacterial-feeding dominance, and the large abundances of those constituents when stimulated to reproduce. By late skeletonization (day 188) community composition, in terms of relative proportions of trophic classes and cp constituencies, had partially recovered to previous levels but still exhibited some enrichment response when compared with initial communities (Fig 3C). It should be noted that by convention, the faunal profile does not include herbivorous taxa in calculations of enrichment or structure indices (EI or SI), as these organisms, while free-living, are plant feeders and therefore not directly subject to fluctuations in microbial food sources [68]. Redundancy analysis (RDA) showed that decomposition stages differed in community membership during decomposition progression, and that community composition at the end of the study (day 317) was fundamentally different from both initial conditions and controls (Fig 4A).

During the community composition shift occurring on day 15 (active decay) as bacterial feeding populations changed from general representation across B1-4 taxa to a dominance by B1 taxa, a succession of B1 enrichment opportunists were observed. Of the seven B1 taxa present, representing five families, only three taxa, representing two families, responded strongly to decomposition enrichment. Diplogasteridae exhibited a sharp and distinct maximum abundance on day 15. Following that, Rhabditidae, of which approximately 30% belonged to the genus *Pelodera*, peaked on day 40. Rhabditidae (and *Pelodera*) enrichment markedly decreased by day 188. No other B1 taxa responded appreciably to enrichment products (S1 Fig). After day 40, *Acrobeloides* (B2), which did not exhibit early response to decomposition enrichment, began to increase in abundance, and this enrichment trend continued to the end of the study. *Pseudacrobeles* (B2), *Plectus* (B2), and *Prismatolaimus* (B3), all taxa with notable abundances in control and initially unimpacted soils, were not appreciably affected by decomposition products or microbial fluctuations throughout the study (S1 Fig).

Fungal-feeding populations, with the exception of *Filenchus* (F2), were notably absent in core soils during the early phases of decay (days 0–6). *Aphelenchoides* (F2), *Boleodorus* (F2), *Ditylenchus* (F2), and Tylencholaimidae (F4) all increased in abundance beginning on day 15 and continuing for the remainder of the study, with final sample enrichments surpassing those of controls. In contrast, *Filenchus* (F2) abundances decreased on day 15 with the deposition of decomposition products, and with the exception of day 21, remained suppressed for the duration of the study. *Diphtherophora* (F3) and *Ecphyadophora* (F4) remained largely unaffected throughout the study (S1 Fig).

Omnivorous taxa were generally found in low abundance in core soils and demonstrated varied response to nutrient enrichment. Of the more *r*-selected taxa in this group, *Achromadora* (O3), mixed Dorylaimida (O4), and *Eudorylaimus* (O4) all exhibited suppression at the onset of active and advanced decay (days 15–21) and dropped below detection by day 78 at the beginning of skeletonization. *Achromadora* abundances recovered by day 110, remaining enriched with respect to control abundances for the remainder of the study. Dorylaimida was enriched at the very end of skeletonization. *Eudorylaimus* peaked in days 153–188 in late skeletonization. All other taxa exhibited no distinct trends other than generalized suppression throughout day 78, and reappearance beginning in day 110 (S1 Fig).

Predators, with the exception of *Seinura* (P2), *Tripyla* (P3), *Clarkus* (P4), and Nygolaimidae (P5), were low in abundance and no overall response to decomposition products was observed. *Seinura*, uncommon in controls, increased by an order of magnitude in late-decomposition soils on days 153 and 317. *Tripyla* was suppressed between days 40–188, spanning late advanced decay through mid-skeletonization, and recovered by day 250. *Clarkus* increased in abundance between days 6–21 (bloat through early advanced decay) and again on day 153 in skeletonization. *Nygolaimidae* abundances fluctuated beginning on day 15, remaining suppressed from days 110–250, and increasing by day 317 (S1 Fig).

### Nematode beta diversity—Community structure in interface (0–1 cm) samples

**Fig 5:**
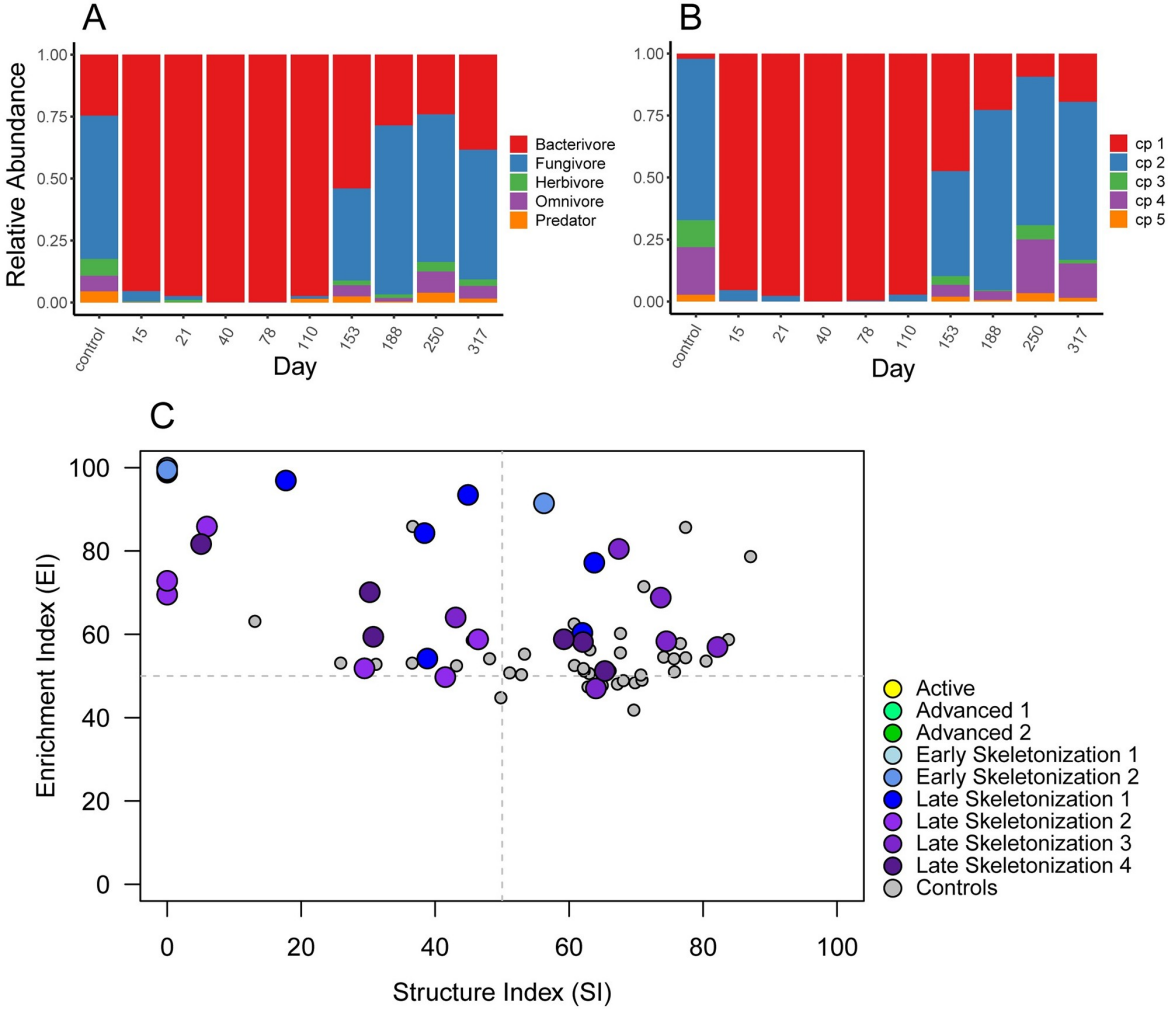
Temporal changes in nematode community composition and functional diversity in interface (0–1 cm) soils. Relative abundances of (A) trophic groups, (B) cp-classes, and (C) functional diversity analysis for interface (0–1 cm) soils.

Interface soils from control sites were dominated by fungivores. While composition of decomposition-impacted soils from initial and bloat stages were unknown for interfaces, samples from the onset of active decay (day 15) contained high relative abundances of bacterial feeding taxa that subsequently increased, reaching maximal abundances in late advanced decay through early skeletonization (days 40–110). After day 110, relative abundances of bacterivores decreased. In contrast with 0–30 cm cores, interface community composition at study day 317 generally resembled those of control samples from a trophic standpoint (Fig 5A). Analogous to patterns observed in deeper soils, fluctuations in cp-class composition mirrored those found in trophic groups; a shift occurred from control communities predominantly composed of bacterial and fungal representatives of cp-2 class taxa, to one composed almost entirely of cp-1 class taxa in days 15–110. This was followed by an increase in cp-2 through cp-5 class taxa during the skeletonization process (Fig 5B). Compared to core samples, interface communities exhibited higher relative abundances of bacterial feeders, principally cp-1 taxa, during active decay through late skeletonization (days 15–110). The communities remained enriched in the faunal profile (EI = 100, SI = 0) from advanced decay 2 (day 40) through late skeletonization 1 (day 153) corresponding to the reduction of alpha diversity. Similar to cores, by late skeletonization (day 188) interface communities had only partially recovered to previous levels (Figs 4B and 5C). Overall community change contribution in core and interface throughout the entire decomposition process shows that degree of impact is larger in interfaces than in cores (Fig 4C).

Beginning in active decay (day 15) populations of Rhabditidae and Diplogastridae (B1) began to increase. Rhabditidae abundances peaked twice, first on day 21 and secondly on day 78, corresponding to early advanced decay and early skeletonization. Both peak abundances exceeded those observed in cores by at least an order of magnitude. *Pelodera* were exceptionally abundant in interfaces, twice that of the remainder of the members of family Rhabditidae, which was opposite of the pattern observed in core samples. As with core samples, no other notable enrichment or suppression was observed for any other B1 taxa. Of the B2- taxa, *Acrobeloides and Plectus* exhibited suppression between days 40–110, corresponding to the transition between late advanced decay and through early skeletonization. All other bacterial-feeding taxa were suppressed between days 15–153 corresponding to the onset of active decay through late skeletonization (S2 Fig).

*Aphelenchoides* (F2), *Boleodorus* (F2), *Ditylenchus* (F2), Tylencholaimidae (F4), *Lelenchus* (F2) and *Nothotylenchus* (F2) were suppressed to undetectable levels beginning on or shortly after day 15 and recovered between days 78–153 during the early part of skeletonization. This pattern was similar to those observed in core soils; however, cores on days 0–6 exhibited undetectable or near-undetectable abundances prior to an increase on day 15; day 0–6 data is not available for interface soils. *Filenchus* (F2) dropped below control abundances by day 21 and below detectable levels for days 78–110 and remained below control abundances for the duration of the study (S2 Fig).

In contrast to core samples, community shifts observed in omnivores in the interfaces was sharply delineated with strong suppression evident from day 15 through 110 in all taxa except *Thorneella* (O4), *Thornia* (O4), and *Ecumenicus* (O5); these latter constituted rare taxa, observed only in controls or in one impacted sample. General community recovery began by day 153, marking the beginning of late skeletonization, with variable abundances in all taxa except Dorylaimida which increased in abundance until the end of the study (S2 Fig).

Predators, with the exception of *Seinura* (P2), *Clarkus* (P4), and Nygolaimidae (P5), were found in low abundance and exhibited no overall response to decomposition products in interface soils. As seen in cores, *Seinura* enrichment occurred in interfaces as well; here the enrichment occurred on day 110, and the enrichment factor was by two orders of magnitude with respect to controls. *Clarkus*, the most abundant predator in control samples, was suppressed between days 15–110 and exhibited recovery to roughly half of control abundances by the end of the study. *Nygolaimidae* abundances were suppressed in days 40–78, after which recovery occurred (S2 Fig).

### Nematode indicator taxa

The partitioning of long cores into shorter segments for depth-stratified studies has been shown to select against rare taxa, and in assessing any given taxon for indicator status, rare taxa, while of interest, are not necessarily of primary concern, especially due to potentially low shifts in abundance with high statistical variability. Taxa that exhibited clear patterns of change in both core and interface soils that may be useful as indicators of decomposition stages were identified. These candidate indicator taxa largely fell into four distinct categories: B1 enrichment only, early decay suppression with late decay enrichment, suppression only, and non-B1 enrichment only.

Three taxa were identified as B1 enrichment indictors in both core and interface soils. Diplogastridae exceeded threshold values on days 15 and 40 in cores, and days 15 through 110 in interfaces. Both *Pelodera* and Rhabditidae exceeded threshold values on days 40 through 110 in both cores and interfaces. Rhabditidae additionally exceeded threshold values beginning in day 15. Two taxa met indicator criteria in one soil depth but not in both: Bunonematidae and *Diploscapter* both met enrichment criteria in interfaces on single dates, days 79 and 188, respectively, but did not meet those criteria in cores. No B1 taxa demonstrated suppression at any point in the study.

Taxa that exhibited early decay suppression followed by late decay enrichment in both cores and interfaces included *Acrobeloides* (B2) and *Aphelenchoides* (F2), and *Boleodorus* (F2). *Acrobeloides* met suppression criteria on day 40 in cores; this period began earlier and was extended in interfaces (days 15, and 40–110). Enrichment occurred on day 78 in cores, day 21 in interfaces, and days 153 through 317 in both depths. *Aphelenchoides* suppression occurred in cores between days 0 through 6 and 21 through 40, and in interfaces for an extended period, days 15 through 110. Enrichment occurred on day 78 in cores, day 21 in interfaces, and throughout days 153–371 in both depths. *Boleodorus* exhibited suppression intermittently until day 188 in cores, and from days 15 through 153 in interfaces. Enrichment occurred only on study day 317. Both *Prismatolaimus* (B3) and Tylencholaimidae (F4) met criteria for suppression (days 0 through 78) and subsequent enrichment (day 153) in cores, but general suppression and only enrichment to recovery levels in interfaces. *Plectus* (B2) satisfied initial suppression criteria in both cores (days 78 through 110) and interfaces (days 15 and 40 through 188), however satisfied subsequent enrichment criteria only in cores on day 153, and could thus be considered to belong to two separate indicator categories based on sampling depth.

*Filenchus* (F2), which had the largest resident population in control soils, was the only taxon to exhibit suppression-only behavior in both soil depths, falling below threshold values on days 40, 110, and 188 in cores, and throughout days 15–188 in interfaces. *Filenchus* did not exhibit enrichment but did partially recover to background levels by the end of the study. Both *Ceratoplectus* (B2) and *Pseudacrobeles* (B2) satisfied suppression-only criteria for the entire study in interfaces but not in cores. Conversely, *Lelenchus* (F2) satisfied suppression criteria in interfaces only.

The dorylaimids (*K*-selected organisms) were the only group to exhibit non-B1 enrichment in both cores and interfaces, this occurring after day 250. Seven taxa from a variety of cp-classes met criteria as indicators in specific soil depths, however, most did so for very brief periods. Three taxa exhibited single-day enrichment in cores: *Ditylenchus* (day 317), *Ecphyadophora* (day 78), and *Eudorylaimus* (day 188). Two others met single-day enrichment criteria in interfaces: *Aporcelinus* (day 317) and *Chiloplacus* (day 78). The two taxa that met enrichment criteria for multiple consecutive sampling time points were both found in interfaces: *Nothotylenchus* between days 78 and 110, and *Seinura* with abundance increases by an order of magnitude between days 110 and 153.

## Discussion

The decomposition of vertebrate remains is a unique process in comparison with the decomposition and nutrient cycling associated with plant material. Vertebrate remains are composed of protein-rich tissues, have a low C:N ratio, and are considered a high-quality resource, thus the breakdown of these products has shown to differ considerably in pattern and resource inputs into the soil creating a “hotpot” pulse event rather than the sustained release of nutrients characteristic of decaying plant matter [69]. While nematode community composition has been evaluated over a variety of agricultural and field conditions, few studies have explored responses associated with vertebrate hotspots [24,26–28,35–43].

Our study investigated temporal changes of nematode communities underneath decomposing vertebrate carcasses in order to discern successional characteristics and functional diversity changes associated with hotspot enrichment. We additionally identified potential indicator taxa associated with decomposition progress. During the course of this experiment, we elected to add a second sampling depth consisting of soil interfaces: The 0–1 cm upper-most layer of soil in immediate contact with decomposition products. This allowed for direct comparison between nematode community responses resulting from close proximity to the enrichment source (i.e. the decomposing carcass), and those deeper in the soil that would be responding to changes in the soil environment due to the enrichment. We focused on the upper layers of soil because it has been demonstrated that approximately 70% of nematodes exist in the top 20 cm of soil. Nematode community composition at a local scale is most commonly associated with available food sources, both horizontally and vertically [28,47,48]. Therefore, we hypothesized that the highly enriched interface soils and deeper soils might harbor unique nematode community assemblages and/or successional patterns. Indeed, we found that enrichment response was more pronounced in interface samples than in deeper soils by an order of magnitude, and that both trophic response and cp-classes mirrored each other within respective strata. This mirroring is partly based upon the manner in which cp-classes are constructed (B1 = bacterial feeder with high enrichment response, B2 = bacterial feeder with moderate enrichment response, and so forth). We also found that as succession progressed, a limited number of taxa exhibited unique enrichment or suppression patterns.

### General successional patterns

Our study demonstrated that nematode abundances, alpha and beta diversity, and functional diversity responded immediately to the introduction of decomposition products into the soil during active decay (day 15) in both cores and interfaces, with the strongest effects observed in interface samples (Figs 2, 3C, 4C and 5C, S1 Table). Overall, there do not appear to be markedly different community compositions between the two soil depths, and taxa that are present in each depth appear to be positively or negatively affected by decomposition products in a consistent manner (S1 and S2 Figs). The greatest differences in response between the soil depths appear to arise from the abundance magnitudes, particularly those of the cp1/B1 bacterial feeders. This is readily apparent by contrasting the consistency found between beta and functional diversity response (Figs 3 and 5) with the variability observed in the RDA composite that compares the response of the two soil depths (Fig 4C). Fig 4C illustrates that while community composition between the two depths overlaps throughout the study, the magnitude of response within those depths, as evidenced by distance from centroids, indicates that the response to decomposition products is exaggerated in interfaces in comparison with cores (Figs 3A, 3B, 4C, 5A and 5B). This exaggerated response in interfaces likely originates from a particularly high concentration of microbial abundances in the uppermost soil layer. In comparison, a 30 cm soil column would require infiltration of decomposition products and food sources, some of which could be lost in the soil aggregate structure.

The nematode community during active decay was dominated by bacterial-feeding enrichment opportunists (cp-1/B1) composed almost exclusively of two families, Rhabditidae and Diplogasteridae, which was consistent both in terms of general behavior and composition with previous reports assessing the effects of vertebrate pig decomposition on nematode community structure in a temperate forest in Switzerland [24]. Elevated abundances in both cores and interfaces in our study persisted throughout late skeletonization (day 153) for a total period of 138 days. In agricultural systems, the addition of organic amendments is typically associated with an increased abundance of *r*-selected bacterial-feeding nematodes (cp-1/B1) as a result of stimulated bacterial growth [32].

In our study, internal carcass temperatures diverged from ambient air temperatures immediately prior the onset of active decay (day 13) and remain elevated until early advanced decay (day 254). Thermogenesis associated with decomposing carcasses is well-known, and is considered primarily attributable to the large maggot masses that develop during active decay [70]. This generates maximum temperatures that can range from (27–50°C) based upon size and density of the maggot mass that is present [70]. Elevated internal carcass temperatures were also reported by Payne (1965) [54], as well as from a similar experiment performed by Keenan et al. (2018) [56] at a site located approximately 500 m from our study [2,54]. Keenan et al. (2018) [56] found that internal core temperatures and soil temperatures exceeded ambient temperatures for a period of 4 days during active decomposition. Payne (1965) [54] performed a decomposition experiment using baby pigs and reported that during bloat throughout advanced decay, internal carcass and soil temperature means all exceeded ambient air temperatures. In another pig decomposition study, Szelecz et al. (2016) [24] reported elevated nematode abundances beginning on day 15 of their study followed by a sharp reduction in abundances beginning on day 22; they did not measure temperature, but proposed that this might be related to thermal stress due to decomposition processes. In composting systems, Steel et al. (2010) and (2013) found sharp reductions in nematode abundances when compost temperatures exceeded 70°C, but they demonstrated that Rhabditidae and *Aphelenchoides* spp. could survive during this period and proliferate once the heat peak passed and temperatures fell below 30°C [71]. Temperatures exceeding 25°C have adverse reproductive effects upon many rhabditids, and temperatures of 24.6°C exceed the thermal tolerance of *Rhabditis cucumeris*, specifically [72]. We observed elevated internal and soil temperatures associated with maggot proliferation but did not see discernible evidence of temperature-induced suppression of nematode abundance. Evidence from compost systems (Steel et al., 2010, 2013) [71] suggests that the duration or magnitude of high temperatures in our study was not long enough to affect nematode reproduction. Another possible contributing factor to the lack of temperature suppression may be due to the fact that the beavers in this study weighed approximately 20 to 23 kg, whereas the pigs in Szelecz et al. (2016) [24] were larger (28 kg) and may have hosted a larger maggot mass that contributed to enhanced heating.

### Cp-1 response patterns

Not all taxa in the cp-1 class responded to enriched soil conditions at the same time or with similar abundances. For the purposes of this discussion it should be noted that *Pelodera*, a genus within the family Rhabditidae, was counted as an individual taxon due to its prominent presence in these samples. No other genera within the family of Rhabditidae displayed conspicuous abundances, and therefore the remainder were simply combined under the general heading of Rhabditidae. Interface samples exhibited a sequential response to enrichment in which Rhabditidae spp. reacted the most quickly (day 15), followed by *Pelodera* (day 40), with abundances almost double those of the other collective Rhabditidae. Members of Diplogastridae were present throughout this period but with unremarkable abundances in comparison with Rhabditidae. Soil cores, on the other hand, exhibited distinct maxima beginning with a brief spike in Diplogastridae (day 15), followed by simultaneous maxima of *Pelodera* and Rhabditidae (day 40), and in contrast to interface samples, Rhabditidae abundances were approximately twice those of *Pelodera*. Of the seven cp-1 taxa present in these samples, only three (Rhabditidae, *Pelodera*, and Diplogastridae) were found in quantity, and met the criteria for consideration as enrichment indicator taxa across both sampling depths. These findings are consistent with results reported by nematode-specific experiments involving decomposing pigs, compost development, and agricultural systems, as well as generalized eukaryotic sequencing surveys of decomposition that have noted the presence of nematodes amongst other taxa [7,14,15,18,24,44,68]. Szelecz et al. (2016) reported that in pigs decomposing on the ground, the three nematode families with enriched abundances were Rhabditidae, Diplogasteroididae, and Neodiplogasteridae. In organically-enriched agricultural soils and cow dung the three nematode families most often reported in largest abundance are Rhabditidae, Panagrolaimidae, and Diplogastridae [30,32,41]. Amplicon sequencing has consistently reported a robust presence of Rhabditidae in broad 18S surveys. Metcalf et al. (2016) [7] additionally noted the presence of Monhysteridae associated with decomposing humans and Panagrolaimidae with decomposing mice, although in both instances absolute abundances were not reported. It is notable that in all cases members of the family Rhabditidae are consistently associated with a range of organic enrichment types and are found in high abundance on these occasions. The order Diplogastrida (which includes the families Diplogastridae, Diplogasteroididae, and Neodiplogasteridae) are consistently found in studies that involve manual extraction and also exhibit high abundances, suggesting the possibility that molecular studies may be less-sensitive to this particular group of taxa. Interestingly, some Diplogastrids, notably *Pristionchus* spp., have recently been observed enriched in rotting vegetal material (including fruit), expanding on previous reports of their necromenic association with beetles [73]. Thus, there is the possibility that Diplogastrids may be found to be general enrichment opportunists transferred between enrichment patches via phoresy, which could explain our observed Diplogastrid blooms from very low soil control densities. Panagrolaimidae abundances were found to be negligible both in this study and in Szelecz (2016) [24] and its presence was reported intermittently in molecular studies. Taken together this suggests that the family Rhabditidae and the order Diplogastrida offer the most consistently robust response in decomposition hotspot environments.

We also observed that while Rhabditidae was enriched to varying degrees over much of the decomposition process (through day 188), enrichment of other B1 taxa was more short-lived in comparison. For example, *Pelodera* and Diplogastridae abundances were elevated between days 15–110. This result suggests that the presence of Diplogastridae enrichment is constrained to a window (in this case approximately 100 days) representing active decay through early skeletonization. *Pelodera* abundances peak very distinctly on day 40, congruent with peak abundance and diversity impacts in both core and interface soils, thus potentially functioning as a genus-level indicator for this narrow period of time. At family-level taxonomic rank Rhabditidae does not appear to provide further resolution than *Pelodera*, simply due to generalized long-term enrichment. Other free-living Rhabditidae genera, notably *Oscheius tipulae*, have been reported in amplicon sequencing surveys during the period of advanced decomposition [18]. While our study did not differentiate Rhabditidae at the genus level beyond that of documenting the outstanding representation from the genus *Pelodera*, it is worth noting that enrichment responses of genera found within Rhabditidae have varied between studies, decomposing organism, and extraction methodologies, whereas the family Rhabditidae as a whole has been shown over multiple studies to respond very strongly and consistently to decomposition, thus suggesting that the application of genus-level resolution to cp 1 enrichment-opportunistic taxa when tracking decomposition progression is not advisable.

### Cp-2 response patterns

Nematodes occupying the cp-2 class, which includes both bacterial and fungal feeders, generally became more dominant as the populations of cp-1 opportunists declined due to dwindling microbial food supply. In our study, cp-2 taxa displayed mixed reactions to decomposition enrichment. The general opportunists *Aphelenchoides* (F2) and *Acrobeloides* (B2) exhibited maximum abundances during late skeletonization (day 153) in cores, and in interfaces a month later (day 188), increasing in abundance throughout skeletonization. This *Aphelenchoides* enrichment was also observed post-heating in compost systems [71]. *Aphelenchoides* and *Acrobeloides* both satisfy criteria for consideration as suppression indicator taxa at the onset of active decay and through the early phases of skeletonization (days 15–110). Additionally, *Aphelenchoides* and *Acrobeloides* also satisfy enrichment criteria as indicators to frame the transition from early to late skeletonization, so it is possible that this pair of nematodes might be used in tandem to describe a midpoint in the skeletonization process around day 153. While suppression is suggestive of either environmental sensitivity or competition for resources, and conversely an enrichment response is suggestive of increased food availability, further study needs to be done comparing microbial quantities and changes in soil chemistry to see what these decomposition periods directly correspond to.

*Filenchus* (F2) was the most abundant fungivore found in both interface and core control samples. During the early stages of decomposition *Filenchus* abundances sharply decreased in both cores and interface samples, and upon reappearance remained below control levels. This suppression of *Filenchus* in close temporal proximity with Rhabditidae (and *Pelodera*) peak abundances poses questions as to how *Filenchus* responds to the types of soil nutrient enrichment and/or prevailing conditions found in decomposition environments, as well as suggesting its use as a suppression indicator taxon to frame this early decomposition time period. We previously reported low abundances of *Filenchus* in a four-year-old grave [56], suggesting a potential sensitivity to decomposition products. In a controlled heating study of compost, Steel et al. (2013) [71] showed that members of the family Tylenchidae (which includes *Filenchus*) did not survive a simulated heat peak of 60°C, whereas *Aphelenchoides* and Rhabditidae were found to survive this same heating process. While soil temperatures in our study did not approach those found in compost, they do suggest that even slightly elevated temperatures may have had a negative effect upon Tylenchidae. To our knowledge our study constitutes the second observation of an inverse correlation between *Filenchus* and select cp- 1–2 taxa, suggesting either a potential interaction between *Filenchus* and other cp- 1–2 taxa or a sensitivity to physical and/or chemical conditions in the decomposition environment. While it is entirely possible that *Filenchus* suppression could arise as a result of competing ecological niches between cp- 1–2 taxa, studies examining niche partitioning using nematodes are uncommon in natural terrestrial ecosystems, aside from the characterization of nematode assemblages in agricultural, forest, and field settings. Far less is known about the effects of hotspot-type pulse disturbances [74]. Under the conditions associated with this study, fungivores and bacterivores belong to separate trophic groups and thus are not presumed to be in direct competition for the same resources. Furthermore, the very nature of a hotspot-type pulse of resources provides large quantities of nutrient enrichment, and thus competition is likely to be reduced. In light of this, we are proposing that the response of *Filenchus* is driven more by thermal or chemical changes in the decomposition environment than by competitive interactions. In order to fully ascertain the effect of the decomposition environment upon *Filenchus*, however, interface data must be included from the beginning of the study, since by the time interface sampling was included in the sampling scheme, *Filenchus* abundances had already appeared to have diverged from control levels.

*Plectus* (B2) has been proposed as a potential indicator taxon responsive to chemical disturbances and stressed environments, shown to be negatively correlated with chemical fertilization, and positively correlated to organic amendments [41,75]. We previously reported examining nematodes associated with decomposition products in a 4-year old multi-individual grave and found large quantities of *Plectus* within areas of still-decaying human remains [56]. However, in our study with beaver carcasses we observed the opposite: *Plectus* abundances fell below those of controls during the most active stages of decomposition, instead meeting suppression indicator criteria. In light of these new observations, it appears that the decomposition response of *Plectus* is inconsistent and requires further study.

Other taxa exhibited a generalized tendency toward decreased abundances at the onset of active decay and recovery or in some cases reappearance during skeletonization, however few were found with robust abundances or consistent patterns of decrease or increase, and therefore use as indicator taxa would not be compelling. Similarly, Szelecz et al., (2016) noted a general increase over time in the cp-2 class, but explicit partitioning into bacterial and fungal feeders was not discussed [24,44].

*Seinura* (P2) abundances peaked in interfaces on day 110, satisfying non-B1 enrichment indicator criteria during days 110–153 (the transition between early and late skeletonization). While *Seinura* abundances by themselves are not large, the increase of an order of magnitude relative to controls is notable in comparison to response by other taxa. *Seinura* is a predator in the family Aphelenchoididae with an unusually short life span ranging from 3 to 6 days; females can lay a single egg approximately every 2 hours upon achieving maturity [76,77]. This localized enrichment of *Seinura* in conjunction with increasing density of both bacterial and fungal-feeding taxa around day 153 suggests that it might occupy a very specific environmental niche based upon availability of food supply or momentary competitive advantage, thus making it a potential indicator taxon associated with the community recovery period occurring during mid-skeletonization.

### Cp-3 through cp-5 response patterns

In agricultural successional studies, as nutrient enrichment is depleted, community structure shifts toward a greater representation of fungal-feeding taxa and the gradual reappearance of *K*-selected taxa (cp-3 through cp-5); these include predators and omnivores that are shown to be sensitive to environmental perturbation [25,49,78]. In our study, cp class 3–5 taxa in carcass interface samples were strongly suppressed or found to be below detection at the onset of active decay in interfaces and during advanced decay in cores, and collectively recovered during skeletonization. Most taxa were of low enough abundance to be considered rare and potentially subject to sampling bias rendering their utility questionable, and thus did not meet suppression indicator taxon criteria; only *Achromadora* (O3), *Clarkus* (P4), and Dorylaimida (O4) were of sufficient abundance in interfaces to merit study, and their collective reappearance occurred by day 153. Of these, only Dorylaimida met criteria as an enrichment indicator in the final portion of the study.

Of potential additional interest are Tylencholaimidae (F4) and *Prismatolaimus* (B3), which were present in control soils, remained suppressed in interface soils for the duration of the study, but recovered in cores at day 153. Both organisms meet criteria for suppression indicators from active decay through early skeletonization, and by late skeletonization serve as enrichment indicators for core soils. The response in interface soils is similar but less well-defined, particularly in the case of *Prismatolaimus*, thus not making it a robust candidate as an indicator. Given the overall rarity of these *K*-selected organisms, coupled with their environmental sensitivity, it might be preferable to consider functional diversity (cp-class composition or feeding type composition) recovery as a more descriptive diagnostic tool for decomposition progression rather than recovery of individual taxa within the original community membership.

Prior analyses of soil chemistry and microbial ecology in gravesoils have revealed temporal fluctuations in carbon and nitrogen speciation, microbial biomass, and respiration throughout decomposition progress. Keenan et al. (2018) [56], in a recent vertebrate (beaver) decomposition study performed in close physical proximity to our study site, found that during the skeletonization period soil pH, electrical conductivity, respiration (evolved CO_2_), and ammonium (NH_4_^+^-N) returned to near background levels from enrichment during active and advanced decay. Also during skeletonization nitrate (NO_3_^-^N) increased. That study began in July and occupied a warmer and thus shorter time period than our study which began in March, and therefore cannot be directly compared. However, evidence from our study suggests that several characteristic shifts in nematode populations might correspond to these chemical and microbial fluctuations. In our study B1 enrichment opportunist (Rhabditidae, Diplogastridae) abundances reduced markedly by the midpoint of skeletonization, commensurate with increases primarily of *Acrobeloides* (B2), *Aphelenchoides*, Tylencholaimidae (F4), and a brief enrichment of *Seinura* (P2). This may correspond to a reduction of microbial activity and/or biomass, particularly bacterial, as shown by a reduction in soil respiration and bacterial production rates [2,6]. This decrease in respiration and biomass suggests that bacterial abundances in the soil have dropped below levels necessary to sustain B1 reproduction, and thus allowing cp-2 class taxa to proliferate. This subsequent increase in cp-2 taxa during skeletonization also includes the appearance of the first fungal feeders, suggesting that fungal biomass may be increasing during this time. Additionally, high concentrations of ammonium typically found in the soil during the early phases of decomposition may have had a deleterious effect upon the more *K*-selected, and therefore more sensitive, nematode taxa [2,4–8]. This disruption is supported by Tenuta and Ferris (2004) when they demonstrated that cp 4–5 class nematodes have difficulty tolerating both specific ion as well as osmotic effects of multiple nitrogen compounds [79].

## Conclusions

We have confirmed that nematode succession occurs in soils as a result of vertebrate decomposition, and that the pattern is consistent with previous reports from other climates. Surface soils were found to experience the largest impact in abundance, alpha, beta, and functional diversity relative to subsurface (core) soils by up to an order of magnitude. Due to varied abundance responses within nematode colonizer-persister (cp) classes to decomposition-induced soil enrichment we propose a series of eight indicator taxa of mixed family and genus taxonomic rank to describe the progression of decomposition. Enrichment in two families, Rhabditidae and Diplogastridae, were observed during active and advanced decay, concomitant with suppression of the genus *Filenchus*, a fungal feeder normally found in abundance in control soils. Early suppression of a mixture of bacterial and fungal feeding taxa (*Acrobeloides*, *Aphelenchoides*, and Tylencholaimidae) beginning in active decay and continuing through the early portions of skeletonization, followed by enrichment midway through the skeletonization process characterize the transition to later phases of skeletonization. Brief *Seinura* enrichment during this skeletonization transition may serve to further describe this period. Cp-class 3–5 taxa have previously been shown to display sensitivity to chemical disruption of their environment, and we observed their collective reappearance during the latest portions of skeletonization, when only dry remains were present. We have confirmed the robust presence of Rhabditidae, Diplogastridae, Cephalobidae (*Acrobeloides*), and *Aphelenchoides* as indicators associated with decomposition and enrichment environments common to observations reported from a variety of geographic regions, and have introduced *Filenchus*, Tylencholaimidae, and *Seinura* as new indicators that warrant further study.

Our study has revealed that the inclusion of interface sampling with traditional coring methods shows considerable promise as an analytical tool for nematode community studies, particularly in instances where severe chemical disruption and the potential for soil heating exists.

Having demonstrated in a vertebrate model system that nematode succession follows distinct temporal and vertical partitioning patterns, and that indicator taxa are apparent, we confirmed that patterns of nematode populations can outline portions of the decomposition process and thus support and confirm other chemical and biological studies of vertebrate decomposition in terrestrial ecosystems.

## Supporting information

S1 Fig Temporal changes in nematode community composition for core (0–30 cm) soils. Heatmap showing mean abundances of taxa present in the following trophic groups: bacterivores, fungivores, omnivores, and predators. Samples are grouped by increasing cp-class, and cp designations are shown. All bacterial and fungal mean abundances are square-root transformed for scaling.
(TIF)

S2 Fig Temporal changes in nematode community composition for interface (0–1 cm) soils. Heatmap showing mean abundances of taxa present in the following trophic groups: bacterivores, fungivores, omnivores, and predators. Samples are grouped by increasing cp-class, and cp designations are shown. All bacterial and fungal mean abundances are square-root transformed for scaling.
(TIF)

S1 Table Mean nematode abundance and alpha diversity by sampling depth. Summary statistics table of all abundance and diversity data.
(XLSX)

S2 Table Effects of decomposition-impacted soils and time on nematode abundances and alpha diversity. Results of two-way ANOVAs comparing impacted soils (Treatment) and study date (Day), as well as interaction effects.
(XLSX)
