# Supplementary figures and images for: Soil nematode functional diversity, successional patterns, and indicator taxa associated with vertebrate decomposition hotspots
Source: PLoS One. 2020 Nov 4;15(11):e0241777. doi: 10.1371/journal.pone.0241777 (PMC7641364; doi:10.1371/journal.pone.0241777)

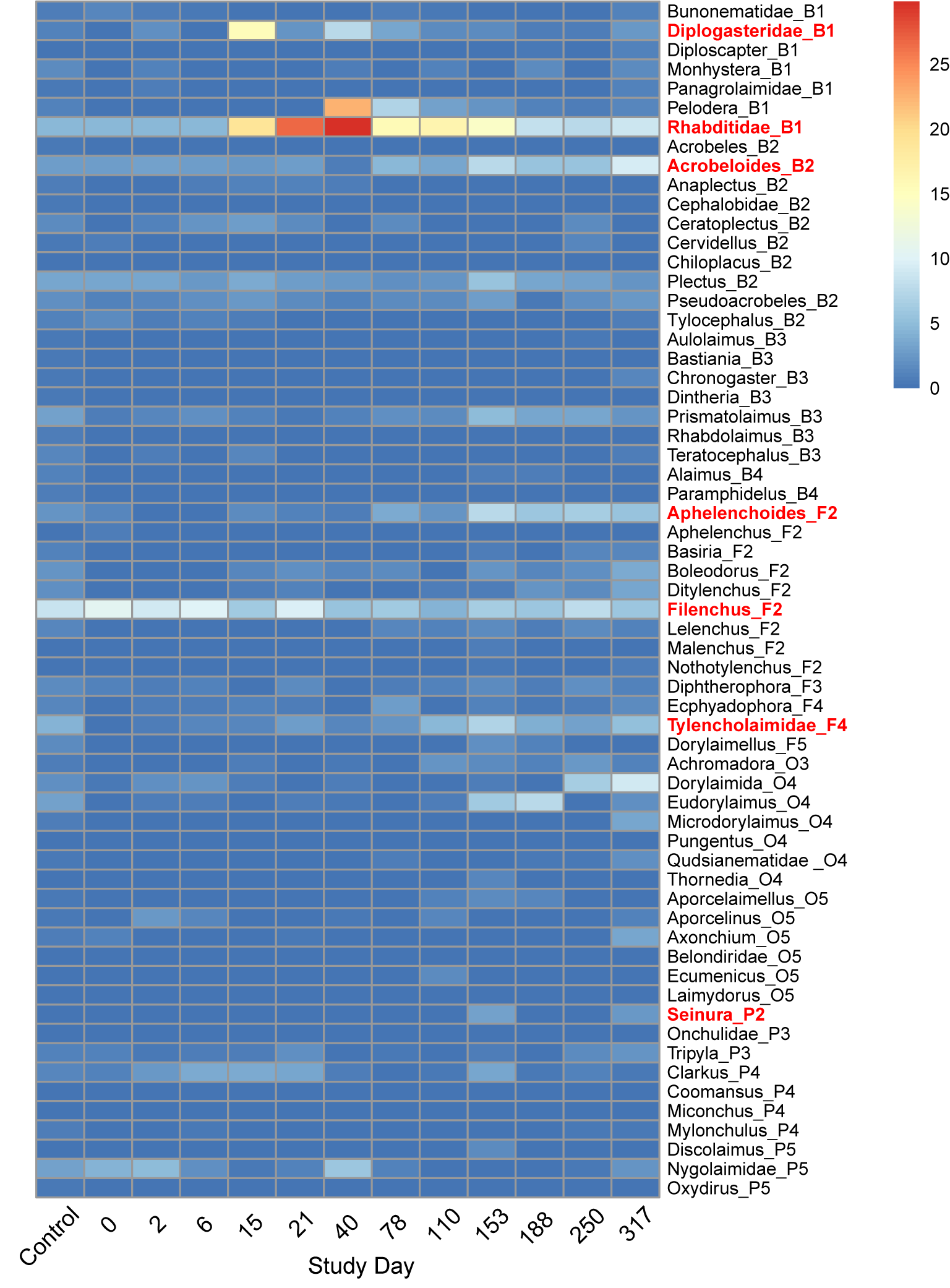

Supplement: S1 Fig — Heatmap showing mean abundances of taxa present in the following trophic groups: bacterivores, fungivores, omnivores, and predators. Samples are grouped by increasing cp-class, and cp designations are shown. All bacterial and fungal mean abundances are square-root transformed for scaling. (TIF) [file pone.0241777.s001.tif]

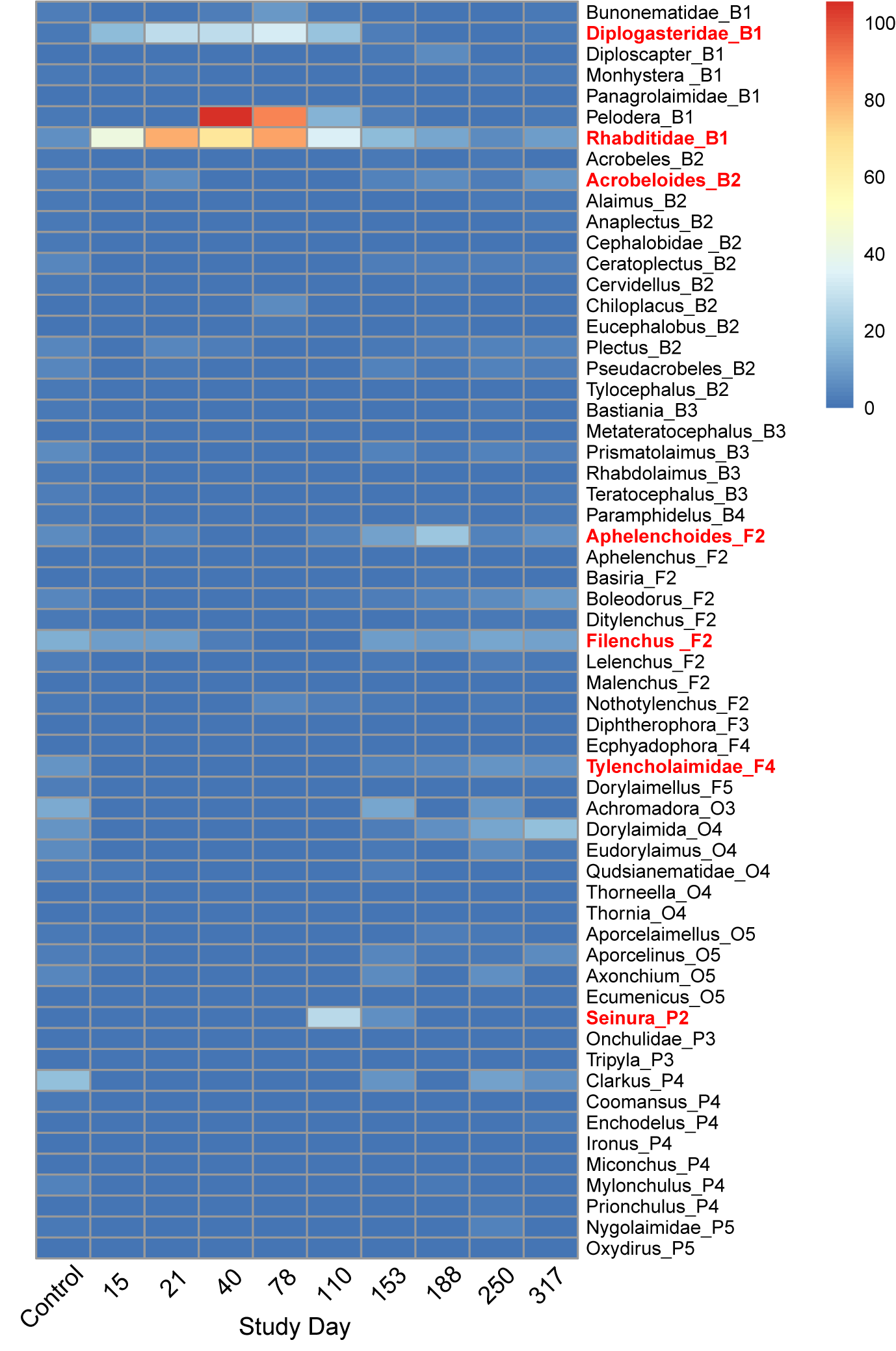

Supplement: S2 Fig — Heatmap showing mean abundances of taxa present in the following trophic groups: bacterivores, fungivores, omnivores, and predators. Samples are grouped by increasing cp-class, and cp designations are shown. All bacterial and fungal mean abundances are square-root transformed for scaling. (TIF) [file pone.0241777.s002.tif]
